# GRP94 is an IGF-1R chaperone and regulates beta cell death in diabetes

**DOI:** 10.1038/s41419-024-06754-y

**Published:** 2024-05-29

**Authors:** Do-Sung Kim, Lili Song, Wenyu Gou, Jisun Kim, Bei Liu, Hua Wei, Robin C. Muise-Helmericks, Zihai Li, Hongjun Wang

**Affiliations:** 1 Department of Surgery, Medical University of South Carolina, Charleston, SC 29425 USA; 2 Microbiology and Immunology, Medical University of South Carolina, Charleson, SC 29425 USA; 3 Pelotonia Institute for Immuno-Oncology, The Ohio State University Comprehensive Cancer Center-James, Columbus, OH 43210 USA; 4 Regenerative Medicine and Cell Biology, Medical University of South Carolina, Charleston, SC 29425 USA; 5 Ralph H. Johnson Veterans Affairs Medical Center, Charleston, SC USA

**Keywords:** Apoptosis, Type 2 diabetes

## Abstract

High workload-induced cellular stress can cause pancreatic islet β cell death and dysfunction, or β cell failure, a hallmark of type 2 diabetes mellitus. Thus, activation of molecular chaperones and other stress-response genes prevents β cell failure. To this end, we have shown that deletion of the glucose-regulated protein 94 (GRP94) in Pdx1^+^ pancreatic progenitor cells led to pancreas hypoplasia and reduced β cell mass during pancreas development in mice. Here, we show that GRP94 was involved in β cell adaption and compensation (or failure) in islets from leptin receptor-deficient (*db/db)* mice in an age-dependent manner. GRP94-deficient cells were more susceptible to cell death induced by various diabetogenic stress conditions. We also identified a new client of GRP94, insulin-like growth factor-1 receptor (IGF-1R), a critical factor for β cell survival and function that may mediate the effect of GRP94 in the pathogenesis of diabetes. This study has identified essential functions of GRP94 in β cell failure related to diabetes.

## Introduction

According to the most recent WHO estimates, about 422 million people worldwide suffer from diabetes. β cell death and dysfunction are hallmarks of all forms of diabetes mellitus [1]. In type 2 diabetes (T2D), β cell death and dysfunction (or beta cell failure) are caused by excessive insulin demand and stress in insulin resistance conditions [2, 3]. Understanding molecular signaling pathways leading to pancreatic β-cell death is critical to the development of rational treatments for diabetes.

Molecular chaperones regulate cellular functions by controlling the maturation of specific protein clients. GRP94 is a molecular chaperone encoded by the *HSP90b1* gene and is the most abundant protein in the ER lumen. GRP94 contributes to ER quality control via chaperoning and folding of its client proteins, interacting with other components of the ER protein folding machinery, calcium storage, and assisting in the targeting of misfolded proteins for ER-associated degradation (ERAD) [4]. Although the upregulation of GRP94 is often used as a hallmark of response to ER stress, significant questions regarding its functional role remain. GRP94 shares similar biochemical features with other HSP90 family members including its domain structure and ATPase activity, but differs in that GRP94 also has calcium-binding ability. As well, GRP94 interactions with co-chaperones are different from other ER chaperones. The clients of GRP94 are restricted to a relatively small number of proteins including immunoglobulin heavy and light chains, insulin-like growth factors (IGF-1 and II), integrins, and Toll-like receptors (TLRs), glycoprotein A repetitions predominant (GRAP) [5], all of which depend on GRP94 for their maturation [6–12]. GRP94 is essential for the development of plants, fruit flies, and mice [10, 13–15]. Homozygous GRP94 KO leads to embryonic lethality in mice. Loss of GRP94 in embryonic fibroblasts leads to a significant decrease in the level of the ER stress-induced spliced form of XBP-1, a downstream target of the IRE1 signaling pathway [16].

GRP94 is abundantly expressed in the pancreas and islets [16]. We previously showed that deletion of GRP94 during embryonic development (in Pdx1^+^ cells) led to increased cell death and reduced β cell mass [17]. Recent genomic studies have shown that a majority of genes involved in the regulation of β cell growth and function during embryonic and fetal development are also associated with increased susceptibility to diabetes [18–22]. Also, GRP94 is demonstrated as essential for proinsulin handling in β cells [23], further suggesting a potential role of GRP94 in β cell death and susceptibility to diabetes. Thus, there is a compelling reason to determine the specific role of GRP94 in the context of diabetes.

IGF-1R is a receptor tyrosine kinase that mediates the action of IGF-1. It binds to IGF-1 with a high affinity and binds to IGF-2 and insulin with a low affinity [24]. Activated IGF-1R is known to be involved in cell growth and survival [25], but regulation of its expression and activation in pancreatic β cells remains largely unknown. In this study, we determined that IGF-1R is a previously unrecognized client of GRP94. GRP94 konck down (KD) or knockout (KO) β cells were more susceptible to apoptosis through the IGF-1R/AKT/Bim axis in vitro when stimulated with stress factors, or in vivo when insulin^+^ β cell-specific GRP94 conditional KO mice were challenged with high fat diet (HFD) to mimic diabetogenic conditions associated with T2D. We conclude that GRP94 plays an essential role in stress-induced β cells, at least in part via regulation of IGF-1R, and that dysfunction of GRP94 may contribute to the development of diabetes and diabetes complications [26].

## Materials and methods

### Mice

Male db/+ and *db/db* mice were purchased from the Jackson Laboratory (Bar Harbor, ME). The mouse strain that contained a floxed GRP94 allele (*Hsp90b1* ^*flox/flox*^*/GRP94*^*flox/flox*^) was described previously [27, 28]. The *Ins1*^*Cre*^ line (B6(Cg)-*Ins1*^*tm1.1(cre)Thor*^/J) was purchased from the Jackson Laboratory. Pancreatic β cell (Ins1^+^)-specific GRP94 conditional knockout (*Ins1*^*Cre*^*; GRP94*^*flox/flox*^, referred to as “KO”) mice were generated by breeding the *Ins1*^*Cre*^ mouse strain with the GRP94^flox/flox^ mice. The *Ins1*^*Cre*^*; GRP94* ^*flox/flox*^ mice were used as the control for the KD mice. For the HFD experiments, 8–10 week-old KO and WT mice were fed HFD (HFD, Research diet Inc, New Brunswick, NJ) (containing 60%, 20%, and 20% of calories from fat, carbohydrate, and protein, respectively) or a normal diet (ND, Research diet INC, New Brunswick, NJ), (containing 10%, 70%, and 20% of calories from fat, carbohydrate, and protein, respectively) for 20 weeks. Blood was obtained from the tail veins of non-fasted mice, and glucose levels were measured using a FreeStyle Lite glucometer (Abbott Laboratories, Abbott Park, IL). Only male mice were analyzed in this study. In all experiments, KO and control were randomized in each group. All procedures and protocols were approved by the IACUC committee of the Medical University of South Carolina.

### Lentiviral infection with the GRP94 shRNA

To generate the GRP94 lentivirus, 293 T cells were co-transfected with GRP94 shRNA (against position 119 of grp94), $\Delta$8.9, and VSV-G plasmids at a ratio of 2:2:1. Forty-eight hours after transfection, lentiviruses were collected and filtered. β cells were transduced by spin infection at 1800 × *g* for 1.5 h at 32 °C. Knockdown of GRP94 in insulinoma βTC3 cells was achieved by 2–3 rounds of shRNA transduction on consecutive days. On day 8, the efficiency of gene knockdown was determined by immunohistological staining or Western blot analysis using the anti-GRP94 9G-10 antibody (Invitrogen, Carlsbad, CA).

### Cell culture and transfection

βTC3 cells were cultured in complete Dulbecco’s modified Eagle’s medium (DMEM) (Hyclone) containing 10% BSA, 100 U/ml penicillin, and streptomycin (Hyclone) in humidified 5% CO_2_, 95% air at 37 °C. Cells were exposed to the following conditions to induce β cell death: 200 μM palmitic acid (Pal), 10 μg/ml tunicamycin, or 1 μM thapsigargin (TG) for the indicated times (Sigma–Aldrich). In some experiments, cells were additionally cultured with 10 μM of selective PI3 kinase inhibitor LY294002, 5 or 10 μM proteasome inhibitor, MG-132, 10 or 50 nM exendin-4 (EX-4, Glucagon-like-peptide-1 analog), 50 ng/ml actinomycin D (ActD, DNA-primed RNA synthesis inhibitor), 1 μM cycloheximide (CHX, eukaryote protein synthesis inhibitor), 50 mM chloroquine (CQ, autophagy inhibitor) and 5 or 10 μg/ml kifunensine (KIF, ER-associated degradation (ERAD) inhibitor) (Sigma–Aldrich). Transfection of βTC3 using the pBABE-bleo IGF-1R Plasmid (Addgen, plasmid #11212) was performed using Lipofectamine 2000 (Invitrogen) according to the manufacturer’s protocol. Stably transformed cells were selected in a medium containing 100 μg/ml Zeocin^TM^ (Invitrogen). In both CON (Santa Cruz, sc-37007) and Bim (Santa Cruz, sc-29803) RNA knocking down experiments, cells were transfected with 10 or 50 nM final concentration of corresponding siRNAs using Lipofectamine 2000 (Invitrogen).

### Mouse islet isolation

Mouse islets were isolated as described [29]. In brief, the pancreas was perfused with 5 ml of 0.6 mg/ml collagenase (Sigma–Aldrich) in Hanks’ buffered saline solution (HBSS, Hyclone) via the pancreatic duct, dissected, and digested at 37 °C for 7 min; the digested pancreas was then passed through a 400-μm wire mesh and rinsed with DMEM (Hyclone) containing 10% (v/v) BSA (Hyclone). Islets were separated by density gradient centrifugation in Histopaque (Sigma). After several washes with DMEM, islets were hand-picked under a dissecting microscope and cultured overnight in DMEM containing 10% BSA, 100 U/ml penicillin, and streptomycin (Hyclone) in humidified 5% CO_2_, 95% air at 37 °C.

### Human islets

Human islets from healthy donors were obtained from Georgetown University. None of the donors had a previous history of diabetes or metabolic disorders. Islet purity was 90%–95%, as assessed by dithizone staining. Islet viability was assessed by the Live/Dead viability/cytotoxicity kit for mammalian cells (Molecular Probe, L-3224). Human islets were cultured in complete Connaught Medical Research Laboratories (CMRL)-1066 (Invitrogen) medium with 5.5 mM glucose containing 10% BSA, 100 U/ml penicillin, and streptomycin (Hyclone) in humidified 5% CO_2_, 95% air at 37 °C.

### Intraperitoneal glucose tolerance test (IPGTT) and C-peptide assay

IPGTT was performed on overnight fasted animals by injecting glucose (2 mg/kg) as described previously [30]. Plasma C-peptide levels were measured using a mouse C-peptide ELISA kit (ALPCO Diagnostics, 80-CPTMS-E01) according to the manufacturer’s recommendation.

### Real-time PCR (RT-PCR) analysis

RNA was extracted from cells and reversely transcribed into cDNA using an RT-PCR kit (Applied Biosystems). Expression of GRP94 mRNA was analyzed using commercially available primers from Applied Biosystems. PCR reactions were performed using the ABI 7700 sequence detection system (Perkin-Elmer, Applied Biosystems) as described previously [31]. Fold changes in gene expression normalized to GAPDH expression were plotted and compared between groups.

### Immunohistochemistry and immunofluorescence

Pancreatic tissues were processed as described [32]. In brief, mouse pancreases were dissected and fixed in 4% formaldehyde at 4 °C for 12 h before embedding in paraffin. Sections of 5 μm were deparaffinized, rehydrated, and incubated with anti-insulin (Thermo Scientific), GRP94 (Enzo), or anti-IGF-1R (Cell signaling) antibodies overnight at 4 °C, followed by fluorescein isothiocyanate (FITC), Cy3- or Cy5-conjugated secondary antibodies (Jackson ImmunoResearch Laboratories). Slides were mounted with VECTASHIELD mount media with 4′6-diamidino-2-phenylindole (DAPI) (Vector Labs). Fluorescence was analyzed using a Zeiss Axio Imager M2 microscope (Carl Zeiss, Inc.), and images were quantified using ImageJ software. Corrected total cell fluorescence (CTCF) of GRP94 fluorescence was quantified using the ImageJ software. The CTCF = Integrated Density – (Area of selected cell X Mean fluorescence of background readings). Quantitative analyses of the pancreatic area were performed on an Olympus BX40 microscope using the Olympus microscopy Image system.

### Morphometric analysis of islet/β cell mass

For morphometric analysis, ten pancreatic sections per mouse spanning the width of the pancreas were analyzed. Pancreatic tissue area and insulin-positive area were determined by computer-assisted measurements using a Zeiss Axio Imager M2 microscope (Carl Zeiss, Inc.), and images were acquired using the ImageJ software. The numbers of islets (insulin-positive aggregates ≥25 µm in diameter) were scored and used to calculate islet density (number of islets per square centimeter of tissue). Mean percentages of β cell fraction per pancreas were calculated as the ratio of insulin-positive area divided by the whole pancreatic tissue area. β cell mass was obtained by multiplying β cell fraction by the weight of the pancreas. Morphometric β cell and islet characterizations were obtained from the analysis of at least 100 islets per mouse.

### Western blot

Islets or βTC3 cells were washed in ice-cold PBS and lysed in lysis buffer containing 20 mM Tris-acetate, 0.27 M sucrose, 1 mM EDTA, 1 mM EGTA, 50 mM NaF, 1% Triton X-100, 5 mM sodium pyrophosphate, 10 mM β-glycerophosphate supplemented with the protease- and phosphatase-inhibitors (Pierce, Rockford, IL, USA). Protein concentrations were determined with the BCA protein assay (Pierce). Equivalent amounts of protein from each treatment group were run on a NuPAGE 4–12% Bis-Tris gel (Invitrogen) and electrically transferred onto PVDF membranes. After 1 h blocking at room temperature using 5% milk (Cell Signaling), membranes were incubated overnight at 4 °C with primary antibodies against AKT (Cell Signaling Technology, #9272 S), ATF-6 (abcam, ab122897), β-Actin (Santa Cruz, sc-492), Bax (Santa Cruz, sc-493), Beclin-1 (Cell Signaling Technology, 3495 P), Bcl-2 (Santa Cruz, sc-69879), Bim (Cell Signaling Technology, #2933 S), CHOP (Santa Cruz, sc-575), cleaved-caspase-3 (Cell Signaling Technology, 9664 P), cleaved-caspase-9 (Cell Signaling Technology, 7237 P), cleaved-PARP (Cell Signaling Technology, 5625 P), eIF2a (Cell Signaling Technology, 9722 S), GRP78 (Santa Cruz, sc-13968), GRP94 (Enzo Lifesciences, ADI-SPA-850), IRE1a (Cell Signaling Technology, 3294 S), IGF-1Rβ (Cell Signaling Technology, 9750 S, 3018 S, 14534 S), IRβ (Cell Signaling Technology, 3020 S) 5625 P LC3A/B (Cell Signaling Technology, 12741 P), p62 (Cell Signaling Technology, 39749 S) p-AKT(S-473) (Cell Signaling Technology, 9271 S), p-AKT(T-308) (Cell Signaling Technology, 4056 S), p-eIF2a (Cell Signaling Technology, 35987 S), p-IGF-1Rβ (Tyr1131)/IRβ (Tyr1146) (Cell Signaling Technology, 3021 S), p-IRE1(S-724) (abcam, ab48187) p-PERK (Santa Cruz, sc-32577) or PTEN (Santa Cruz, sc-7974). Horseradish peroxidase-conjugated secondary antibodies were from Cell Signaling Technology. Signals were visualized using an ECL detection kit (Thermo Scientific, 34096). Each experiment was repeated at least 3 times and the average relative protein expression of genes was quantified using ImageJ software and compared for statistical differences.

### Immunoprecipitation

Cells were washed with cold PBS, and lysed in cold buffer containing 20 mM Tris-HCl (pH 7.5), 150 mM NaCl, 0.27 M sucrose, 1 mM EDTA, 1 mM EGTA, 50 mM NaF, 1% NP-40, 5 mM sodium pyrophosphate, and 10 mM β-glycerophosphate supplemented with proteinase/phosphatase-inhibitors for 30 min on ice. Lysates were centrifuged at 12,000 × *g* for 15 min at 4 °C prior to immunoprecipitation. Immunoprecipitations were carried out by incubating 0.5–1 mg of total lysate with rabbit anti-GRP94 (1:100), or rabbit anti-IGF-1R (1:50) antibodies on a rotator at 4 °C overnight. Immunocomplexes were then captured with Protein A Agarose Fast Flow (Millipore) by rotation at 4 °C for 4 h. After five washes with cold lysis buffer, the immunoprecipitates were resuspended in sample buffer and electrophoretically separated on NuPAGE 4–12% Bis-Tris gels (Invitrogen).

### Protein degradation analysis

βTC3 cells were treated with 50 ng/ml actinomycin D (ActD), or 50 μg/ml cycloheximide (CHX) for 0, 1, 2, and 4 h, and the lysates were subjected to immunoblotting.

### Adenovirus construction and transfection

βTC3 cells were infected with adenovirus expression vectors for Ad5.RIP-GFP (control), Ad5.RIP-CA-AKT (Ad-AKT), Ad5.CMV-wt-AKT1 (control), Ad5. CMV-DN-AKT1 (Ad-DN-AKT) (All kindly provided by Dr. Hongju Wu) at a multiplicity of infection (MOI) of 10 for 4 h. Adenovirus was subsequently washed off with PBS and replaced with a fresh medium with 10% FBS. GSIS or RNA and protein isolation were performed 48 h post-infection.

### Cell viability assessment and proliferation assay

βTC3 cells were treated with β cell death conditions: 200 μM Pal, 10 μg/ml TU or 1 μM TG for the indicated time. Cells were trypsinized and resuspended in a complete medium. Each sample was mixed with Trypan blue solution (0.14% in HBSS, Sigma). Colored (dead) and dye-excluding (viable) cells were counted on a Malassez hemocytometer.

### Statistical analyses

Sample size in each group was chosen based on previous experience in similar studies. Results are expressed as the mean ± standard deviation of the mean of multiple independent experiments, as indicated in figure legends. Statistical analyses were carried out by two-tailed Student’s *t-*test or one-way ANOVA, and *p* < 0.05 was denoted as significant.

## Results

### Time-dependent change of GRP94 expression in islets isolated from the db/db mice

**Fig. 1:**
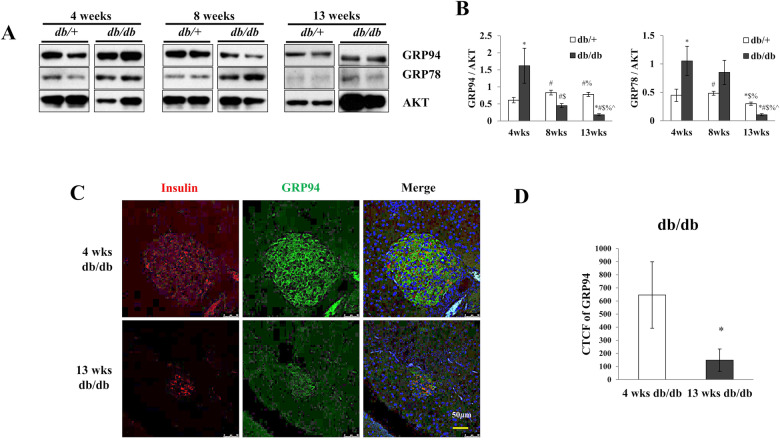
Dynamic change of GRP94 expression in db/db mice at different ages. Expression of GRP94 and GRP78 in mouse islets isolated from 4, 8, and 13-week-old diabetic *db/db* mice and their heterozygous littermates controls by Western blot (**A**) and quantification (**B**). Immunoblots of GRP78, GRP94, and densitometry analyses are shown. ^*^p < 0.05 *versus* 4wks db/+, ^#^*p* < 0.05 *versus* 4wks db/db, ^$^*p* < 0.05 *versus* 8wks db/+, ^%^*p* < 0.05 *versus* 8wks db/db, ^^^*p* < 0.05 *versus* 4wks db/+, one-way ANOVA. **C** Immunostaining for GRP94 (green), insulin (red), and nucleus (blue) in sections from 4 and 13-week-old diabetic *db/db* mice. Scale bar, 50 µm. **D** Histograms show corrected total cell fluorescence (CTCF) of GRP94 fluorescence quantified by ImageJ software. The CTCF = Integrated Density – (Area of selected cell x Mean fluorescence of background readings). *p < 0.05, Student’s *t*-test.

To determine whether GRP94 expression is correlated with the onset and/or the progression of diabetes, we measured protein levels of GRP94 in islets harvested from the *db/db* mice at different ages. At 4 weeks (wks) of age, the expression level of GRP94 was significantly higher in the *db/db* mice compared to age-matched *db/+* control islets (Fig. 1A, B), possibly due to increased stress in the islets. At 8 wks of age, GRP94 protein level was significantly reduced in *db/db* islets compared to control islets (Fig. 1A, B). At 13 wks of age, protein expression of GRP94 was even lower in *db/db* islets compared to control islets as indicated by Western blot (Fig. 1A, B) and immunohistochemistry analysis (Fig. 1C, D). In contrast, protein expression of GRP78, another component of chaperone complex known to regulate unfolded protein response (UPR) signaling through interaction and inhibition of transmembrane ER proteins, GRP78, was higher in *db/db* islets compared to control islets at most time points measured, suggesting a potential compensating mechanism for GRP94 deficiency (Fig. 1A, B). These data suggest a role of GRP94 in islet adaption and compensation (or failure) in obese mice that develop diabetes.

### Characterization of GRP94 KD β cells and KO islets

**Fig. 2:**
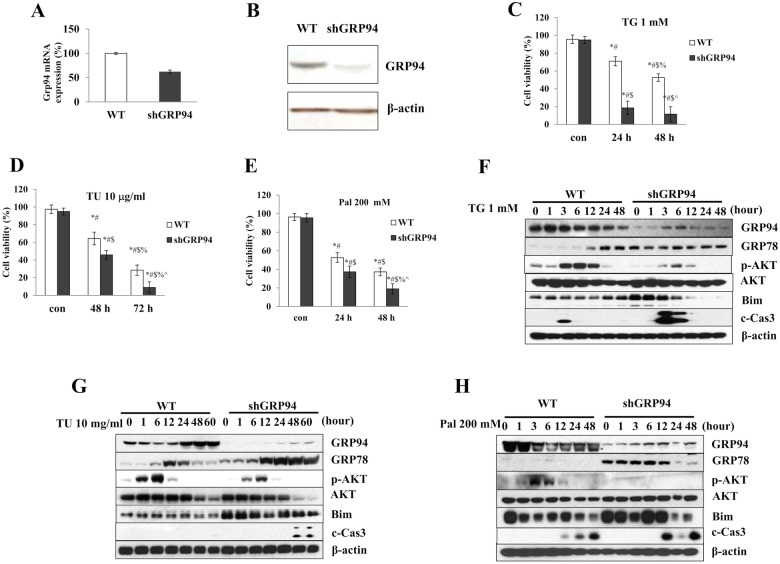
GRP94 KD cells are more susceptible to stress-induced cell death. **A** Relative *GRP94* mRNA expression in WT control and Knockdown cells (shGRP94). **B** Protein expression of GRP94 in WT and KD cells. **C** Viability of WT control (white columns) and KD (black columns) cells after TG (1 μM)^*^p < 0.05 *versus* con WT, ^#^*p* < 0.05 *versus* con shGRP94, ^$^*p* < 0.05 *versus* 24 h WT^, %^*p* < 0.05 *versus* 24 h shGRP94, ^^^*p* < 0.05 *versus* 48 h WT, one-way ANOVA, **D** TU (10 μg/ml)^*^p < 0.05 *versus* con WT, ^#^*p* < 0.05 *versus* con shGRP94, ^$^*p* < 0.05 *versus* 48 h WT, ^%^*p* < 0.05 *versus* 48 h shGRP94, ^^^*p* < 0.05 *versus* 72 h WT^,^ one-way ANOVA, or **E** Pal (200 μM^)*^p < 0.05 *versus* con WT, ^#^*p* < 0.05 *versus* con shGRP94, ^$^*p* < 0.05 *versus* 24 h WT, ^%^*p* < 0.05 *versus* 24 h shGRP94, ^^^*p* < 0.05 *versus* 48 h WT, one-way ANOVA, at different time after treatment. Cell death was measured by trypan blue (*n* = 3). **F**–**H** Protein expression of GRP94^,^ GRP78, p-AKT, AKT, Bim, cleaved Caspase-3 (c-Cas-3), and β-actin in WT control and GRP94 KD cells at indicated times after TG, TU, or Pal treatment as analyzed by immunoblot.

To further elucidate the role of GRP94 in β cells, GRP94 shRNA lentivirus was used to knock down GRP94 expression in the βTC3 insulinoma cell line. Cells infected with the scrambled control virus were used as controls (WT). The mRNA levels of GRP94 in the KD cells were reduced to 62.4% ± 3.2% of WT expression level on day 4 after viral infection (Fig. 2A), and the protein levels were decreased to 18.1% ± 3.5% of WT (Fig. 2B). We also generated GRP94 conditional knockout (KO) mice in which the GRP94 gene was deleted in Ins1^+^ mature β cells by crossing the *Hsp90b1* ^*flox/flox*^ mice with *Ins1-Cre* mice. The β cell-specific GRP94 KO *(Ins1*^*Cre*^*;Hsp90b1*^*flox/flox*^, KO) mice showed normoglycemia and normal development compared to cre control (*Ins1*^*Cre*^) mice. GRP94 expression was specifically reduced in islet cells, confirming tissue-specific gene deletion (Fig. S1B).

We measured the expression of proteins related to ER stress, including GRP78 and its downstream UPR signaling molecules [33] in GRP94 KD β cells and the KO islets. Upregulation of basal levels of GRP78 was evident in the GRP94 KD and KO cells (Fig. S1B). This upregulation was further enhanced by down-regulation of the basal levels of downstream targets of GRP78, inositol-requiring enzyme 1 alpha (IRE1α), phosphor-eukaryotic initiation factor 2α (p-eIF2a), and C/EBP homologous protein (CHOP) (Fig. S1A, B). GRP94 KD cells also had reduced splicing XBP-1 after treatment with TG (Fig. S1C). These results show that β cell-specific deletion of GRP94 triggers expression of GRP78, resulting in down-regulation of UPR signaling which may increase susceptibility to ER stress-induced cell death [16].

### GRP94 KD β cells or KO islets are susceptible to stress-induced cell death

We next measured stress-induced β cell death in GRP94 KD and control cells induced by different stimulators that mimic various diabetogenic conditions that cause ER stress including thapsigargin (TG), tunicamycin (TU), and palmitate (Pal) relevant to type 2 diabetes. The effect of GRP94 knockdown on stress-induced cell death was determined by morphology, cell death staining, presence of cleaved-caspase-3 (c-Cas-3), and activation of other cell death markers. With each treatment, GRP94 KD cells exhibited significantly more death compared to WT cells as indicated by cell viability analysis (Fig. 2, C–E) and the presence of cleaved-caspase-3 (c-Cas-3), a pro-apoptotic protein known to play prominent roles in ER stress-induced cell death (Fig. 2, F–H). We further examined the activation of AKT/Bim and c-Cas-3 in WT and GRP94 KD cells treated with various stimulators. Basal levels of phosphorylated AKT (p-AKT) were markedly reduced in GRP94 KD cells compared to control cells (Fig. 2, F–H). In stressor-treated cells, these were accompanied by further reductions in p-AKT and by increases in expression of cell death markers, including Bim and c-Cas-3 (Fig. 2, F–H), suggesting that GRP94 expression is essential for protecting β cells from stress-induced death through the AKT/Bim signaling pathway. p-AKT levels were much higher in both TG and TU-treated cells for the early time points, which may be due to cell adaption after stimulation [34]. To determine the specificity of the KD, we infected GRP94 KD cells with GRP94 overexpressing or control adenoviruses before stimulation with TG. Overexpression with GRP94 rescued cells from death and confirmed the specificity of GRP94 knockdown (Fig. S2).

### AKT and Bim signalings are critical for TG and TU-induced cell death in GRP94 KD and control cells

**Fig. 3:**
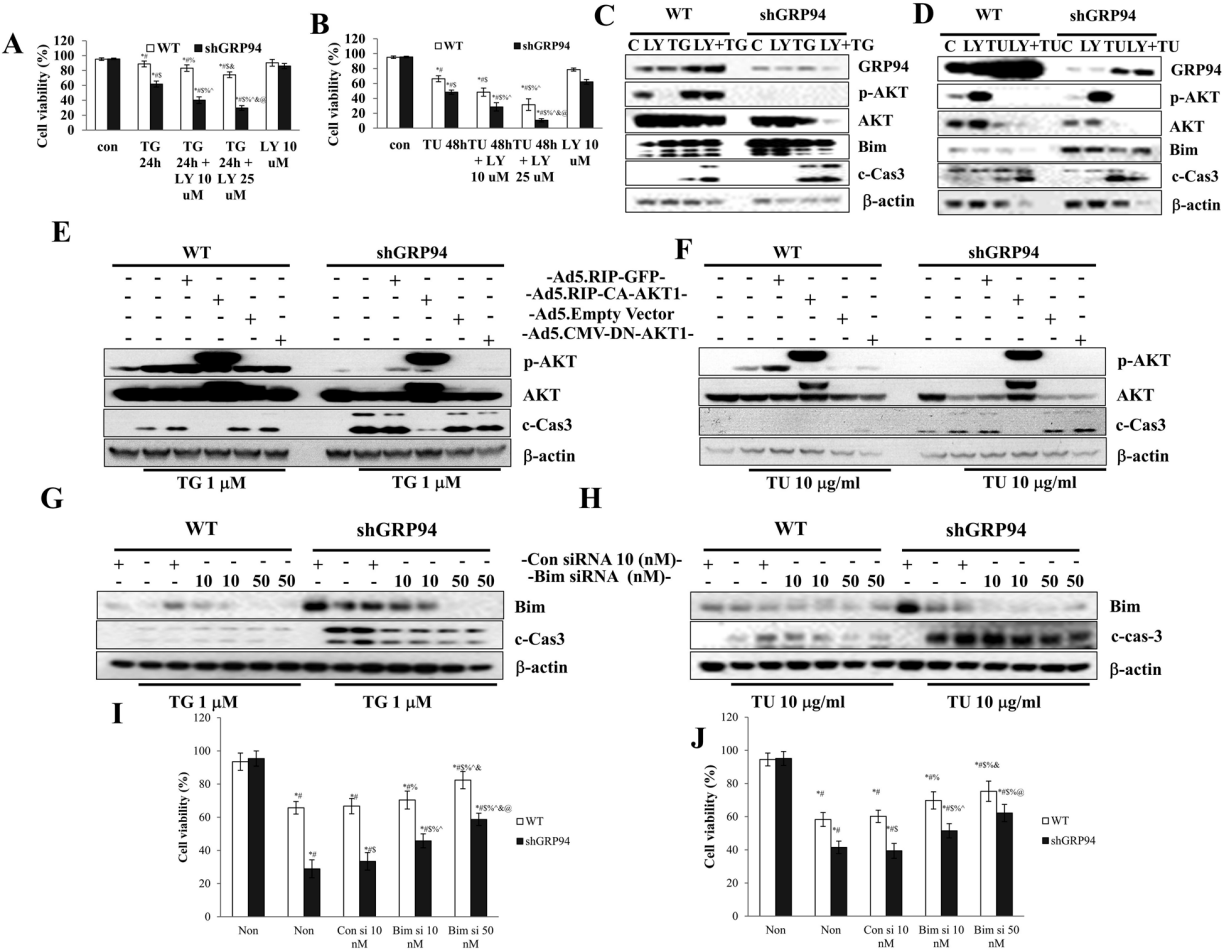
Regulation of AKT/Bim axis protects β cells from TG and TU-induced death. **A**, **B** WT, and GRP94 KD Cells were exposed to LY for 30 min prior to and during the 24 h treatment with TG or 48 h treatment with TU. Cell viability was measured and compared. **A** ^*^p < 0.05 *versus* con WT, ^#^*p* < 0.05 *versus* con shGRP94, ^$^*p* < 0.05 *versus* TG24h WT, ^%^*p* < 0.05 *versus* TG24h shGRP94, ^^^*p* < 0.05 *versus* TG24h + LY10 μM WT, ^&^*p* < 0.05 *versus* TG24h + LY10 μM shGRP94, ^@^*p* < 0.05 *versus* TG24h + LY25 μM WT, one-way ANOVA. **B** ^*^p < 0.05 *versus* con WT, ^#^*p* < 0.05 *versus* con shGRP94, ^$^*p* < 0.05 *versus* TU48h WT, ^%^*p* < 0.05 *versus* TU48h shGRP94, ^^^*p* < 0.05 *versus* TU48h + LY10 μM WT, ^&^*p* < 0.05 *versus* TU48h + LY10 μM shGRP94, ^@^*p* < 0.05 *versus* TU48h + LY25 μM WT, one-way ANOVA, at indicated time after treatment with or without LY. **C**, **D** WT, and GRP94 KD cells were ex*p*osed to LY (10 µM) for 1 h, and treated with or without TG (1 μM) or TU (10 μg/ml). Immunoblots of GRP94, p-AKT, AKT, Bim, and caspase-3 cleavage. **E**, **F** WT and GRP94 KD cells were infected with the indicated adenovirus and then treated with TU or TG. Immunoblots of p-AKT, AKT, and caspase-3 cleavage. **G**–**J** WT and GRP94 KD cells were transfected with control siRNA or Bim siRNA, and then treated with TG for 6 h or TU for 48 h. **G**, **H** Total cell extracts analyzed by immunoblot of Bim and c-Cas-3. **I**, **J** Cell death was determined by trypan blue staining. ^*^p < 0.05 *versus* con WT, ^#^*p* < 0.05 *versus* con shGRP94, ^$^*p* < 0.05 *versus* Consi10nM + TG24h or TU48h WT, ^%^*p* < 0.05 *versus* Consi10nM + TG24h or TU48h shGRP94, ^^^*p* < 0.05 *versus* Bimsi10nM + TG24h or TU48h WT, ^&^*p* < 0.05 *versus* Bimsi10nM + TG24h or TU48h WT, ^@^*p* < 0.05 *versus* Bimsi50nM + TG24h or TU48h WT^,^ one-way ANOVA.

Phosphatidylinositol-3 kinase (PI3K)-AKT signaling is a key regulator of β cell survival and function [35, 36]. AKT is positively regulated by GRP94 in other cell types [37, 38]. We hypothesized that GRP94 is an essential regulator of AKT activation during pancreatic β cell apoptosis based on the fact that GRP94 KD cells exhibited reduced p-AKT expression (Fig. 2F–H). We tested whether treatment with LY294002, a PI3K inhibitor, enhanced β cell death. As shown by the cell viability assay, incubation with LY294002 significantly sensitized WT and GRP94 KD cells to TG- or TU-induced cell death at 48 h after stimulation (Fig. 3A, B). We also measured protein expression levels of GRP94, p-AKT, Bim, and c-Cas-3. TG treatment increased expression of p-AKT, Bim, and c-Cas-3 at 6 h after treatment in WT and GRP94 KD cells (Fig. 3C). LY294002 blocked TG-induced activation of p-AKT, and further increased TG-induced Cas-3 activation (c-Cas-3 levels) in both WT and GRP94 KD cells (Fig. 3C). Treatment with TU also increased expression of c-Cas-3, and decreased expression of p-AKT at 48 h after treatment in both WT and GRP94 KD cells (Fig. 3D). These results indicate that LY294002 inhibited PI3K and AKT leading to lower levels of p-AKT, and potentiating TG- and TU-induced β cell death in WT and GRP94 KD cells. We verified that overexpression of AKT1 by infection with the constitutively active (CA)-AKT1 adenovirus containing an src myristoylation sequence and a GFP-tag, completely inhibited TG- and TU-induced c-Cas-3 expression in both WT and GRP94 KD cells (Fig. 3E, F). Infection with AKT2 or AKT3 did not rescue either WT or GRP94 KD cells from death (not shown). These results suggest that AKT1 is important for β cell survival, and is a downstream signaling molecule in the GRP94 signaling pathway in β cells.

Bim is regulated by the AKT signaling pathway and plays a critical role in cell death [39]. We next determined whether the deletion of Bim rescues GRP94 KD cells from TG- or TU-induced cell apoptosis. We first transfected WT or GRP94 KD cells with two different doses of Bim or control siRNA. Transfection with Bim siRNA led to decreased expression of Bim in both WT and GRP94 KD cells (Fig. 3G, H). Bim deletion led to a dose-dependent reduction of TG- or TU-induced c-Cas-3 production and an increase in cell viability in GRP94 KD cells (Fig. 3I, J). These data show that Bim acts downstream of GRP94, and shows that inhibition of GRP94 further increases TG- and TU-induced β cell apoptosis in a Bim-dependent manner.

### GRP94 is required for membrane expression of IGF-1R

**Fig. 4:**
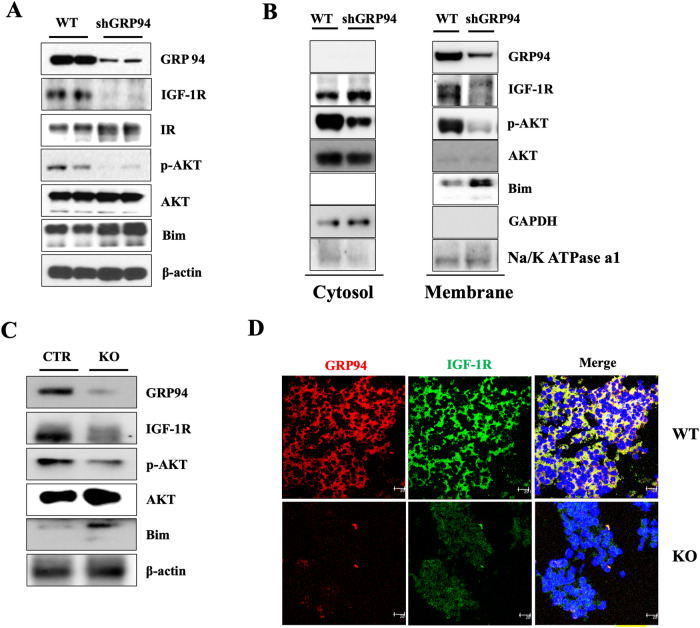
GRP94 is required for membrane expression/maturation of IGF-1R. **A** Immunoblot analysis of GRP94, IGF-1R, IR, p-AKT, AKT, Bim, and β-actin from total lysates of WT and GRP94 KD cells. **B** Immunoblot analysis of GRP94, IGF-1R, p-AKT, AKT, Bim, and GAPDH from cytosol or membrane of WT and GRP94 KD cells. **C** Immunoblot analysis of GRP94, IGF-1R, p-AKT, AKT, Bim, and β-actin in islets harvested from 8-weeks old control or GRP94 KO KO mice. **D** Immunofluorescence analysis of GRP94 (red), IGF-1R (green), and nucleus (blue) in WT or KO mouse islets. Scale bar, 25 µm.

IGF-1 and IGF-2 are clients of GRP94 [10, 11]. IGF-1R mediates the signaling pathway of IGF-1 and IGF-2. We tested whether IGF-1R is also a client of GRP94 in pancreatic β cells by Western blot and immunohistochemistry analyses. Expression of IGF-1R was dramatically decreased in GRP94 KD cells compared to WT cells (Fig. 4A). This was further confirmed by cytosol and membrane fraction immunoblot analysis in which we saw that membrane expression of IGF-1R was lost in GRP94 KD cells but not in WT cells (Fig. 4B). We measured the expression of molecules downstream of IGF-1R signaling, and found that GRP94 KD cells had reduced phosphorylation of AKT but increased Bim expression (Fig. 4A). Since there is crosstalk between insulin receptor (IR) and IGF-1R [40], we also measured the expression of IR in GRP94 KD cells. We found that there was no difference in the expression of IR between WT and GRP94 KD cells (Fig. 4A). In addition, the expression of mature IGF-1R was also significantly reduced in GRP94 KO islets compared to controls (Fig. 4C, D). These data show that GRP94 expression is required for membrane expression of IGF-1R in β cells.

**Fig. 5:**
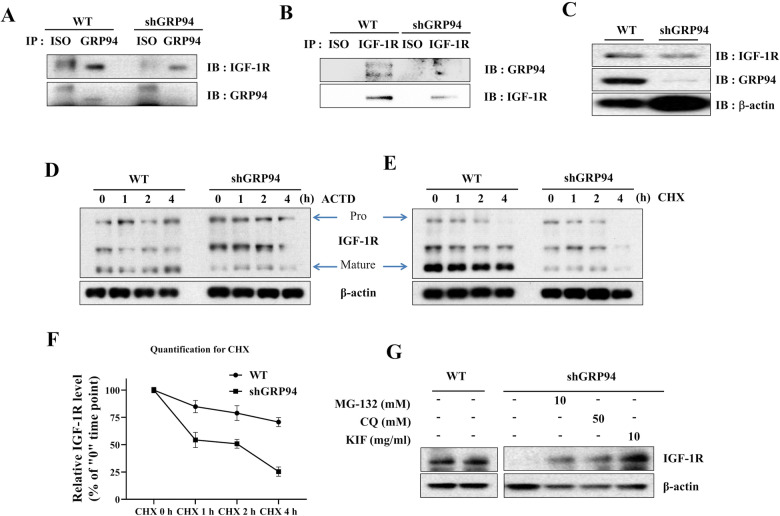
GRP94 interacts with IGF-1R, and is a critical chaperone for the expression/maturation of IGF-1R. **A** Immunoblot of IGF-1R and GRP94 following immunoprecipitation of GRP94 from lysates of WT or GRP94 KD cells. **B** Immunoblot of IGF-1R and GRP94 following immunoprecipitation of IGF-1R from lysates of WT or GRP94 KD cells. **C** Immunoblots of IGF-1R in WT and GRP94 knockdown cells. **D** WT and GRP94 knockdown cells were treated with actinomycin D (5 μg/ml, **D**) or cycloheximide (100 μg/ml, **E**) for indicated periods of time. After stimulation, the lysates were collected and IGF-1R levels were assayed by immunoblot. **F** The degradation/half-life after CHX treatment was calculated as (value at 1 h - value at 4 h)/Value at 1 h. Degradation rate was calculated based on three independent experiments using ImageJ quantification. Pro-IGF-1R degraded faster than the mature form of IGF-1R. **G** Immunoblot analysis for IGF-1R expression in WT and GRP94 KD cells after treatment with proteasome inhibitor (MG-132), autophagy inhibitor (CQ), or ERAD inhibitor (kifunensine, KIF).

We next determined whether there is a direct interaction between GRP94 and IGF-1R by co-immunoprecipitation (IP) experiments using the anti-GRP94 antibody as the pull-down antibody and the anti-IGF-1R or the anti-GRP94 antibodies as immunoblotting antibodies in WT and GRP94 KD cells. We found that GRP94 interacted with IGF-1R during the co-IP. KD cells exhibited significantly reduced binding to IGF-1R compared to WT (Fig. 5A). The association between GRP94 and IGF-1R was further confirmed by reciprocal immunoprecipitation when the anti-IGF-1R antibody was used as the pull-down antibody and the anti-GRP94 as the immunoblotting antibody (Fig. 5B), which established the direct interaction between IGF-1R and GRP94 in β cells. The presence of GRP94 and IGF-1R was also detected in the immunoprecipitated samples (Fig. 5C).

We further assessed whether GRP94 regulated IGF-1R expression at transcriptional, translational, and/or post-translational levels by measuring IGF-1R protein expression in WT and GRP94 KD cells in the presence or absence of actinomycin D (ACTD), a reagent that blocks RNA transcription, or cycloheximide (CHX), a eukaryotic protein synthesis inhibitor. We found that treatment with ACTD did not impact the expression levels of mature IGF-1R in either WT or GRP94 KD cells (Fig. 5D). However, treatment with CHX led to faster IGF-1R protein degradation in GRP94 KD cells (Fig. 5E). The calculated half-life of IGF-1R was 5.95 ± 0.3 h in WT cells but only 2.05 ± 0.5 h in GRP94 KD cells (Fig. 5F). These results suggest that GRP94 modulates the protein stability of IGF-1R.

IGF-1R is degraded through proteasomes, lysosomes, and the ER-associated protein degradation (ERAD) pathways [41, 42]. To probe whether decreased IGF-1R expression in GRP94 KD cells was due to increased degradation of IGF-1R, we treated WT and GRP94 KD cells with inhibitors of protein degradation pathways involving proteasome (using MG-132), lysosome (using chloroquine, CQ) or the ERAD pathway (using Kifunensine, KIF), and measured IGF-1R expression in WT and KD cells. We found that IGF-1R expression in GRP94 KD cells could be restored by each of the three inhibitors (Fig. 5G), suggesting that IGF-1R was degraded by ERAD through proteasomal and lysosomal pathways in the absence of GRP94.

### IGF-1R/p-AKT/Bim signaling is blocked in the GRP94 KD cells

**Fig. 6:**
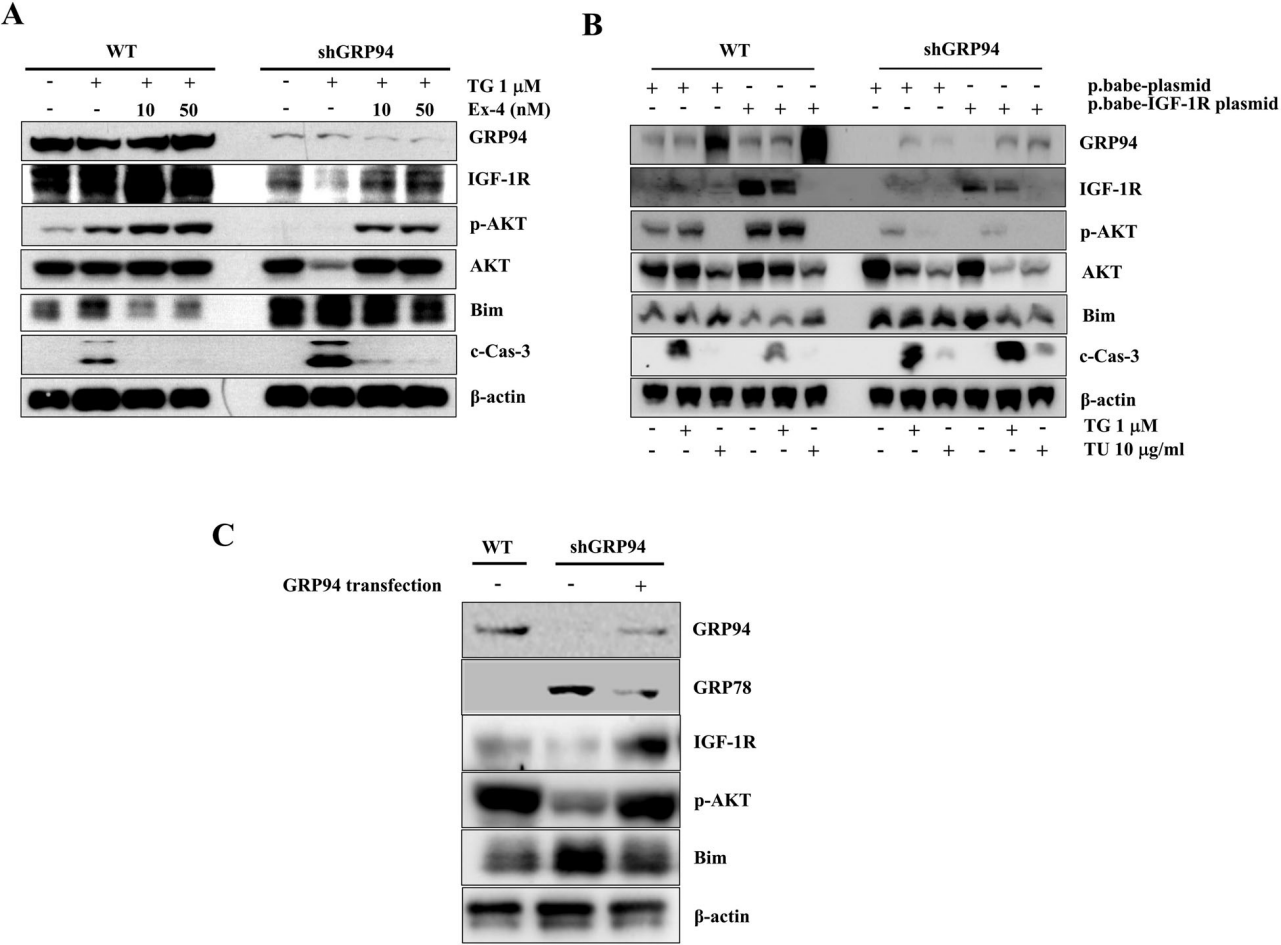
Treatment with Exendin-4 or overexpression of IGF-1R or GRP94 protects β cells from TG-induced apoptosis. **A** WT and GRP94 KD cells were treated with 1 μM TG in the absence or presence of 10 nM or 50 nM Exendin-4 for 6 h. Total cell extracts were analyzed by immunoblot for GRP94, IGF-1R, p-AKT, AKT, Bim, c-Cas-3, and β-actin. **B** WT and GRP94 KD cells were transfected with control plasmid (p.babe plasmid) or IGF-1R overexpression (p.babe-IGF-1R) plasmid and then treated with TG for 6 h or TU for 48 h. Total cell extracts were then analyzed by immunoblot for GRP94, IGF-1R, p-AKT, AKT, Bim, c-Cas-3, and β-actin. **C** GRP94 KD cells were transfected with GRP94 WT plasmid. Total cell extracts were then analyzed by immunoblot for GRP94, IGF-1R, p-AKT, Bim, and β-actin.

Glucagon-like peptide-1 (GLP-1) has been shown to protect β cells against apoptosis by enhancing the activity of the IGF-2/IGF-1R autocrine loop [43]. To determine whether there was a link between GRP94 and the IGF-1R/p-AKT/Bim signaling pathway, we measured the expression of IGF-1R, p-AKT, Bim, and c-Cas-3 after treatment with exendin-4 (Ex-4), a GLP-1 agonist, in WT and GRP94 KD β cells in the presence or absence of stimulators. Treatment with Ex-4 reduced β cell death and c-Cas-3 activation induced by various stimuli (TG, TU). Treatment with exendin-4 restored IGF-1R and p-AKT expression and reduced Bim and c-Cas-3 expression in TG-treated WT and GRP94 KD cells (Fig. 6A and Fig. S3). These data suggest that GRP94 contributes to β cell survival via the IGF-1R/p-AKT pathway.

We determined whether forced expression of IGF-1R rescued GRP94 KD cells from TG- or TU-induced cell death. Treatment with TG or TU led to increased apoptosis as indicated by increased Bim and c-Cas-3 protein expression in both WT and GRP94 KD cells. In contrast, forced expression of IGF-1R in WT cells reduced TG- and TU-induced Bim and c-Cas-3 protein expression, indicating that overexpressing of IGF-1R in WT but not in GRP94 KD cells protected cells from ER stress-induced apoptosis (Fig. 6B). As expected, IGF-1R was only overexpressed in WT cells and not GRP94 KD cells, further confirming that GRP94 is required for IGF-1R maturation. IGF-1R couldn’t be induced in WT or KD cells treated with TU due to its suppression of N-glycosylation needed for IGF-1R maturation. These data also further confirmed that IGF-1R is the downstream effector of GRP94 in β cells and that IGF-1R plays a critical role in β cell death. To assess whether the expression of GRP94 was sufficient for IGF-1R expression, we transfected GRP94 KD cells with a plasmid encoding GRP94 [12] and measured the expression of GRP94 and IGF-1R/p-AKT/Bim. We also found that overexpression of GRP94 led to increased expression of IGF-1R/p-AKT, and reduced expression of Bim in the KD cells, suggesting that expression of GRP94 is not only required but is sufficient for IGF-1R expression in β cells (Fig. 6C).

### GRP94 deletion aggravates β cell death from HFD-induced diabetes

**Fig. 7:**
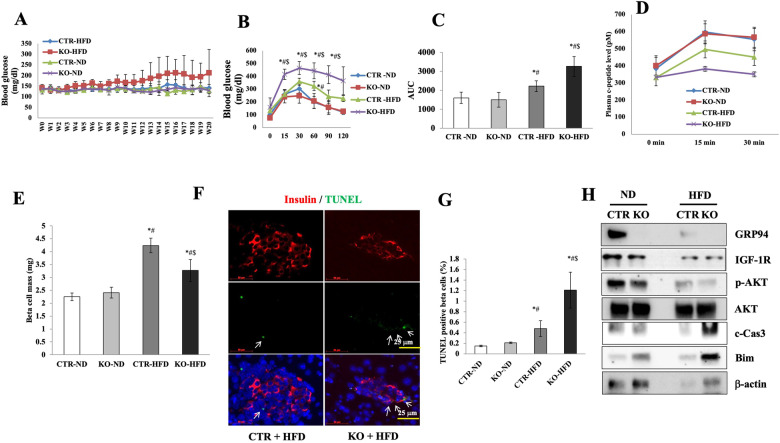
GRP94 deletion increases β cell susceptibility to HFD-induced β cell death and diabetes progression. GRP94 KO (*n* = 12) and Cre control (*n* = 12) mice fed normal diet (ND) or HFD for 20 weeks. Random-fed blood glucose levels (**A**), blood glucose levels during an IPGTT (**B**), and area under the curve (AUC) during an IPGTT after HFD (**C**). ^*^p < 0.05 *versus* control-ND, ^#^*p* < 0.05 *versus* KO-ND, ^$^*p* < 0.05 *versus* control-HFD, one-way ANOVA. **D** C-peptide secretion during an IPGTT measured before (0 min), 15 min, and 30 min after glucose injection. ^*^p < 0.05 *versus* control-ND, ^#^*p* < 0.05 *versus* KO-ND, ^$^*p* < 0.05 *versus* control-HFD, one-way ANOVA. **E** β cell mass was analyzed in ND-fed and HFD-fed mice. Ten pancreatic sections from each individual mouse (*N* = 4 per group) were analyzed. ^*^p < 0.05 *versus* control-ND, ^#^*p* < 0.05 *versus* KO-ND, ^$^*p* < 0.05 *versus* control-HFD, one-way ANOVA. **F** Fluorescence analysis from triple staining for TUNEL, insulin, and DAPI. White arrows point to TUNEL^+^ cells. **G** Histogram shows percentages of TUNEL-positive β cells in each group. Scale bar, 50 µm. ^*^p < 0.05 *versus* control-ND, ^#^*p* < 0.05 *versus* KO-ND, ^$^*p* < 0.05 *versus* control-HFD; one-way ANOVA. **H** Protein expression in mouse islets isolated from all 4 treatment groups at week 21. Immunoblot shows relative protein expression of GRP94, IGF-1R, p-AKT, AKT, c-Cas-3, and β-actin. ^*^P < 0.05.

We investigated the impact of GRP94 deletion on diabetes development in a mouse model of obesity and insulin resistance mimicking T2D. We fed GRP94 KO and control mice a regular diet (ND) (*n* = 12) or an HFD (*n* = 12) for 20 weeks. In general, HFD control and HFD GRP94 KO mice gained more weight than ND mice. β cell-specific disruption of GRP94 did not affect weight gain or food intake in either group (Fig. S4A, B). HFD feeding increased non-fasting blood glucose levels in GRP94 KO mice, but the difference from controls did not reach statistical significance (Fig. 7A). At 20 weeks of HFD feeding, HFD control and HFD GRP94 KO mice showed impaired glucose disposal and increased glucose AUC during the IPGTT compared to ND mice (Fig. 7B). HFD GRP94 KO mice exhibited more severely impaired glucose disposal than HFD control mice manifested by markedly elevated blood glucose levels at most time points, increased AUC and lower plasma C-peptide levels (*n* = 6, Fig. 7, B–D). These data suggest that GRP94 expression in β cells is a mediator of resistance to HFD-induced β cell loss.

Consistent with the metabolic phenotype of ND GRP94 KO mice, HFD GRP94 KO mice exhibited a reduced compensatory β cell mass relative to HFD control mice after HFD feeding (Fig. 7E). To assess the mechanisms by which GRP94 deletion affects β cell mass during HFD feeding, we examined β cell apoptosis in islets by TUNEL assay. The percentage of TUNEL^+^ positive β cells was significantly higher in HFD GRP94 KO mice than in all control groups (Fig. 7F, G). Cell death was further confirmed in isolated islets by the presence of c-Cas-3 by Western blot analysis. HFD control islets showed lower expression of IGF-1R, p-AKT, and increased Bim compared to ND controls. In contrast, significantly increased c-Cas-3 expression was observed in HFD GRP94 KO islets compared to islets from ND GRP94 KO, ND control, or HFD control mice at 20 weeks of age (Fig. 7H). HFD KO islets exhibited the lowest expression of IGF-1R, p-AKT, and the highest expression of Bim among all groups (Fig. 7H). These data are consistent with our in vitro observations indicating increased death in the GRP94 KD cells compared to control cells when challenged with different stressors. We did not observe any difference in pancreatic β cell proliferation among groups (not shown). These results suggest that β cell-specific GRP94 deletion leads to decreased pancreatic β cell mass and decreased islet density predominantly due to increased β cell apoptosis in response to HFD stimulation. GRP94 is a critical regulator of β cell death by regulating the IGF-1R/AKT/Bim signaling pathway.

## Discussion

Stress-induced pancreatic β cell adaption and compensation (or failure) is one of the major features of type 2 diabetes. Understanding molecular mechanisms responsible for β cell death holds promise for improving the treatment of diabetes. In this study, we identified a novel cytoprotective effect of GRP94 in stress-induced pancreatic β cells during the development of T2D. Most importantly, we provide evidence that IGF-1R is a previously unrecognized client of GRP94 in β cells and that GRP94 mediates IGF-1R-associated prosurvival effects. Furthermore, we show that GRP94 KD or KO β cells are more susceptible to stress-induced apoptosis through the IGF-1R/AKT/Bim axis. Understanding the role of GRP94 in stress-induced β cell death may contribute to developing novel therapeutic approaches to treat diabetes.

Components of the unfolded protein response (UPR) play a dual role in β cells, acting as beneficial regulators under physiological conditions, as well as triggers of ER stress and β cell apoptosis in pathological conditions contributing to the development of diabetes [44–46]. The UPR increases the biosynthetic capacity of secretory pathways through the upregulation of ER chaperone and foldase expression [45]. A recent study shows that diabetes can be reversed by controlling UPR activation and ER stress [47], which necessitates the study of the roles of molecules involved in this process. GRP94 is a molecular chaperone involved in cell survival regulation, and plays an important role in UPR and ER stress-induced cell death [48–50]. Although GRP94 is one of the most abundantly expressed glycoproteins in the ER, and is highly expressed in the pancreas [51], its roles in β cell development [17] and proinsulin folding [23] have only recently been reported by others and us. It is likely that these roles of GRP94 have gone unrecognized because GRP94 is more selective than many other ER chaperons; however, the basis of this selectivity remains unknown [51].

Previous studies have shown that GRP94 protein levels decreased during ER stress-induced apoptosis [52, 53]. We found that expression levels of GRP94 were significantly increased and then decreased as diabetes progressed in the db/db mice. These findings suggest that GRP94 has a cytoprotective function and the reduction of GRP94 expression is associated with the progression of diabetes in the T2D mouse model. It is worth noting that GRP94 mRNA was overexpressed in patients with type 2 diabetes [23]. These results suggest that cells have the ability to adjust the ER chaperone balance in response to the depletion of a key chaperone such as GRP94.

The prosurvival effect of GRP94 was tested in vitro by challenging GRP94 KD β cells with diabetogenic factors including TG, TU, or palmitate. In each condition, GRP94 KD β cells showed increased susceptibility to stimuli that mimic the causes of T2D. This suggests that GRP94 is a common component in the multiple signaling pathways leading to β cell death. Indeed, deletion of GRP94 correlated with reduced p-AKT expression, caspase-3 activation, and increased cell death in etoposide-treated Jurkat cells [50]. These in vitro data support the prosurvival role of GRP94 observed in β cells in vivo in the db/db mice.

A key finding of this study is that we identified IGF-1R as a client of GRP94. IGF-1R plays a critical role in β cell survival via the IGF-2/IGF-1R autocrine loop [43]. In this pathway, IGF-2 binds IGF-1R, stimulating its auto-phosphorylation of tyrosines, which leads to the recruitment and activation of PI3K. The kinase AKT is subsequently activated by phosphorylation and protects β cells from stress-induced death [54, 55]. We found that GRP94 protein modulates the stability of IGF-1R, a key factor required for the maturation and membrane expression of IGF-1R. The fact that cell death can be prevented by overexpression of GRP94 alone, but not by overexpression of IGF-1R alone suggests that GRP94 is required for the protective effect of IGF-1R.

Since insulin and IGF-1 can both activate IGF-1R signaling, we examined whether cells treated with insulin or IGF-1 before TG treatment differed in the expression of IGF-1R and TG-induced cell death. Treatment with insulin or IGF-1 increased phosphorylation of insulin receptor (IR)/IGF-1R (the antibody recognizes both), but failed to restore IGF-1R levels or rescue cells from TG-induced apoptosis in KD cells (not shown). These findings exclude the possibility that GRP94 is involved in insulin and IGF-1 signaling pathways in β cells in TG-induced cell death. Furthermore, GRP94 does not regulate IR expression, although IGF-1R and IR share more than 50% sequence homology and over 80% homology in the intracellular kinase domain [56]. This further confirms that IGF-1R and IR mediate distinct cellular and physiological functions. Therefore, our data show that GFP94 is required and sufficient for membrane expression and signaling transduction of IGF-1R and is the chaperone of IGF-1R.

The AKT pathway plays an essential function in regulating β cell survival. The IGF-1R/AKT pathway is aberrantly activated in multiple cancers and promotes proliferation, survival, growth, and metabolism [57]. AKT initiates phosphorylation of various substrates and positively regulates insulin transcription, secretion, and β cell growth and survival [35, 36]. Gene transfer of active AKT1 by an infectivity-enhanced adenovirus increases β cell survival in vitro [58]. Previous studies suggest that GRP94 regulates AKT phosphorylation [37, 38]. This is consistent with our finding that p-Akt, and total AKT, were both reduced in KD cells compared to control cells before and after ER stress induction. AKT isoforms 1–3 are encoded by separated genes and have unique activation modes and many distinct functions [59]. We found that overexpression of Akt1 but not of Akt2 or Akt3 could rescue GRP94 KD cells from cytokine-induced cell death. These data offer additional evidence that AKT isoforms mediate distinct functions. Thus, our data show that activation of the PI3K-AKT pathway in β cells abrogated diabetogenic β cell apoptosis, especially under ER stress in GRP94 KD cells, whereas suppression of PI3K-AKT signaling induced increased β cell apoptosis compared to control cells. These data suggest that AKT is down-regulated by GRP94/IGF-1R in β cells, and indicate that AKT mediates the effects of GRP94 in β cells.

Activated AKT can promote cell survival by phosphorylating and inhibiting or activating various pro- or anti-apoptotic factors, including Bim, Bad, and Bid [60–62]. Bim plays a vital role in mitochondrial-dependent apoptosis, and its induction leads to cell death in different cell types [63, 64]. Bim proteins are expressed in a wide variety of tissues but are most prominently expressed by cells of hematopoietic origin. Previous studies have shown that glucose and ribose toxicity-induced β cell apoptosis requires the participation of Bim, Puma, and Bax, but not Bid, Noxa, or Bak [65]. Interestingly, Bim mediates the death of insulin receptor substrate 2 deficient β cells [66]. The present experiments demonstrated that silencing Bim significantly reduced β cell apoptosis in both the WT and GRP94 KD cells, indicating that Bim is an essential mediator of β cell apoptosis induced by GRP94 depletion/IGF-1R deficiency.

β cell mass reflects a dynamic balance between β cell growth and death. An inadequate expansion of β cell mass to compensate for increased insulin demand, followed by the eventual loss of β cells due to apoptosis, is a hallmark of diabetes [2, 67]. The prosurvival effects of GRP94 were observed in HFD-fed mice. Long-term HFD feeding led to a detrimental impact on β cell function and insulin sensitivity and further contributed to glucose intolerance and T2D [68]. β cell-specific GRP94 KO resulted in aggravated glucose intolerance, reduced insulin secretion, and reduced β cell mass, whereas insulin sensitivity was unaffected. Thus, deletion of GRP94 aggravates β cell death in the HFD mouse model. One limitation with the HFD mouse model is that we only measured GRP94 expression level in islets from WT and GRP94 KO mice at 20 weeks after feeding HFD or chow diets in which reduced GRP94 expression was observed in islets from HFD-WT compared to Chow-WT mice. Therefore, we did not know if GRP94 expression was increased before its decrease as part of the compensation response seen in the islets from db/db mice (Fig. 1).

GRP94 KO and IGF-1R KD mice showed average growth and development. Deletion of IGF-1R caused hyperinsulinemia and glucose intolerance without changing β cell mass. Thus, it will be interesting to determine if IGF-1R KO mice exhibit a phenotype similar to GRP94 KO mice concerning β cell death when challenged with HFD. We do not exclude the possibility that GRP94 increased cell death was caused by altered ER quality control after GRP94 deletion [17] since Grp94-deficient cells showed decreased Ire1α and PERK activity, two branches of unfolded protein response (UPR) critical for ER homeostasis and cell fate. It is well established that Ire1 or PERK deficiency impairs β cell function and growth [69, 70]. Moreover, GRP94 is essential for proinsulin handling, whose impairment in GRP94-deficient beta cells causes β cell dysfunction and ER stress.

Recent development of molecular tools and functional assays has expanded our knowledge of the spectrum of clients that rely on GRP94 activity, but it remains unclear how the chaperone binds these clients, or what aspect of folding is impacted. Our next step will be delineating GRP94 binding to and modulation of IGF-1R maturation. Taken together, our findings indicate that GRP94 plays a critical role in β cell survival and that IGF-1R is an unrecognized client of GRP94.

### Supplementary information

Supplemental data

Uncropped Western blots

## Data Availability

The datasets generated during and/or analyzed during the current study are available from the corresponding author upon reasonable request.
