# Supplementary material for: GRP94 is an IGF-1R chaperone and regulates beta cell death in diabetes
Source: Cell Death Dis. 2024 May 29;15(5):374. doi: 10.1038/s41419-024-06754-y (PMC11137047; doi:10.1038/s41419-024-06754-y)
Supplement: Supplementary file 1 — Supplemental data [file 41419_2024_6754_MOESM1_ESM.docx]

Do-sung Kim, et al. GRP94 is an IGF-1R Chaperone and Regulates Beta Cell Death in Diabetes

**Supplemental Figures:**

**

**

**Fig. S1. Expression of UPR proteins in β cells and in mouse islets. (A)** Immunoblots of GRP94, GRP78, IRE1α, p-eIF2α, eIF2α, CHOP, ATF6-a, and β-actin in WT control and GRP94 KD cells. (B). Immunoblots of GRP94, GRP78, IRE1α, p-eIF2α, eIF2α, CHOP, ATF6-α, and β-actin in isolated control and GRP94 KO islets. (C) RT-PCR analysis shows expressions of un-splicing and splicing isoforms of xbp-1 transcripts in WT and GRP94 KD cells.

**

**

**Fig. S2. Overexpression of GRP94 protects β cells from TG-induced apoptosis.** Immunoblots of GRP94 and c-Cas3 in KO cells transfected with control (over con) or GRP94 overexpressing adenovirus (Over GRP94) after treatment with TG.

**

**

**Fig. S3. Treatment with Exendin-4 rescues KD β cells from stressor-induced apoptosis.** Treatment with Exendin-4 partially restored expression of IGF-1R and rescued GRP94 KD cells from (A&B) TG or TU -induced cell death. Immunoblots of c-Cas-3 and β-actin in indicated cells and treatments (D& E).

**

**

**Fig S4. Characterization of β-cell specific GRP94 mice.** (A) Body and (B) average weekly food intake of ET and KO mice fed ND or HFD. CTR-ND: WT mice fed normal chow; CTR-HFD: WT mice fed HFD; KO-ND: GRP94 KO mice fed normal chow; KO-HFD: GRP94 KO mice fed HFD.
