# Supplementary material for: GRP94 is an IGF-1R chaperone and regulates beta cell death in diabetes
Source: Cell Death Dis. 2024 May 29;15(5):374. doi: 10.1038/s41419-024-06754-y (PMC11137047; doi:10.1038/s41419-024-06754-y)
Supplement: Supplementary file 2 — Uncropped Western blots [file 41419_2024_6754_MOESM2_ESM.pptx]

## Slide 1
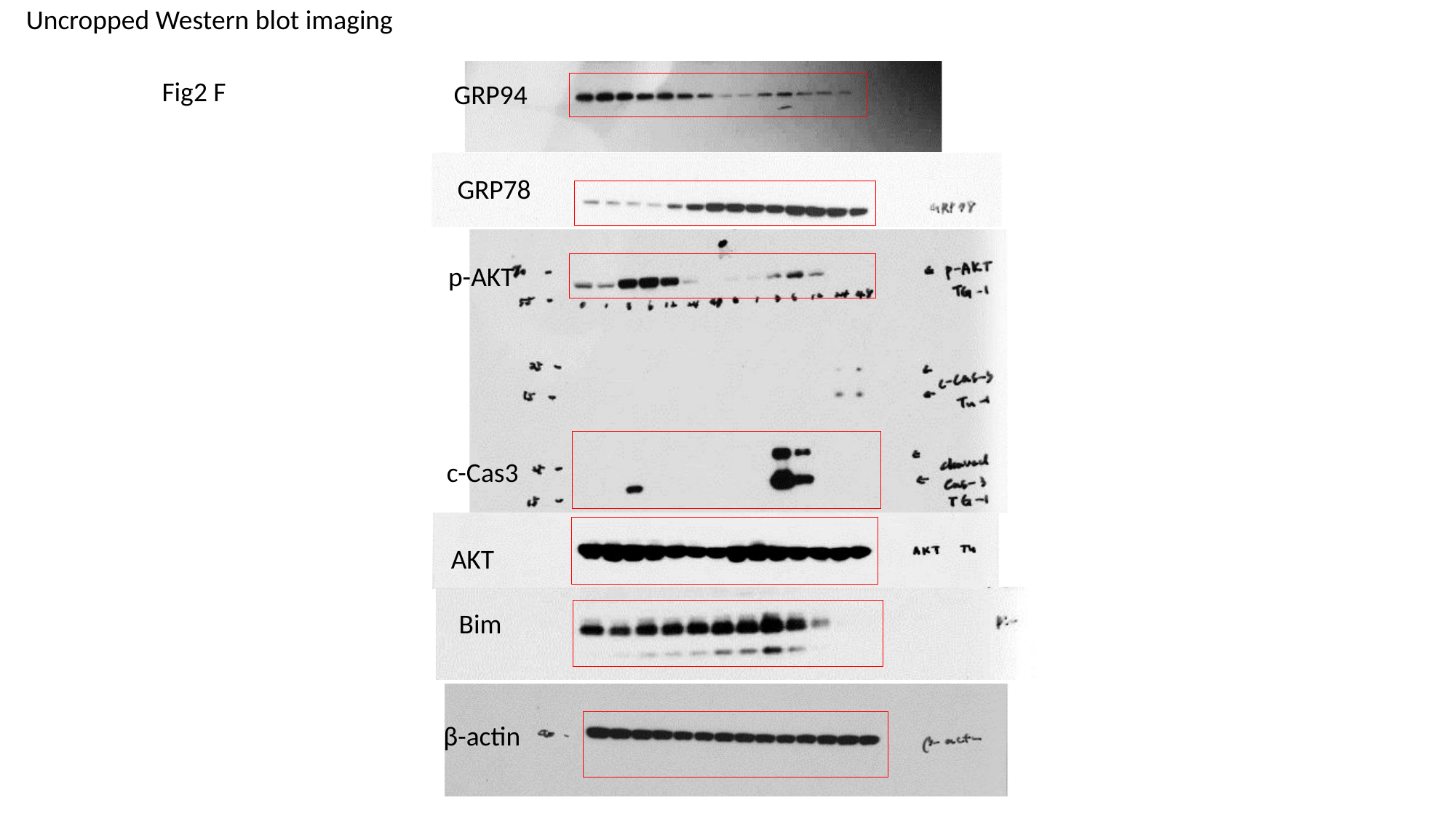

Uncropped Western blot imaging
GRP94
Fig2 F
GRP78
p-AKT
c-Cas3
AKT
Bim
β-actin

## Slide 2
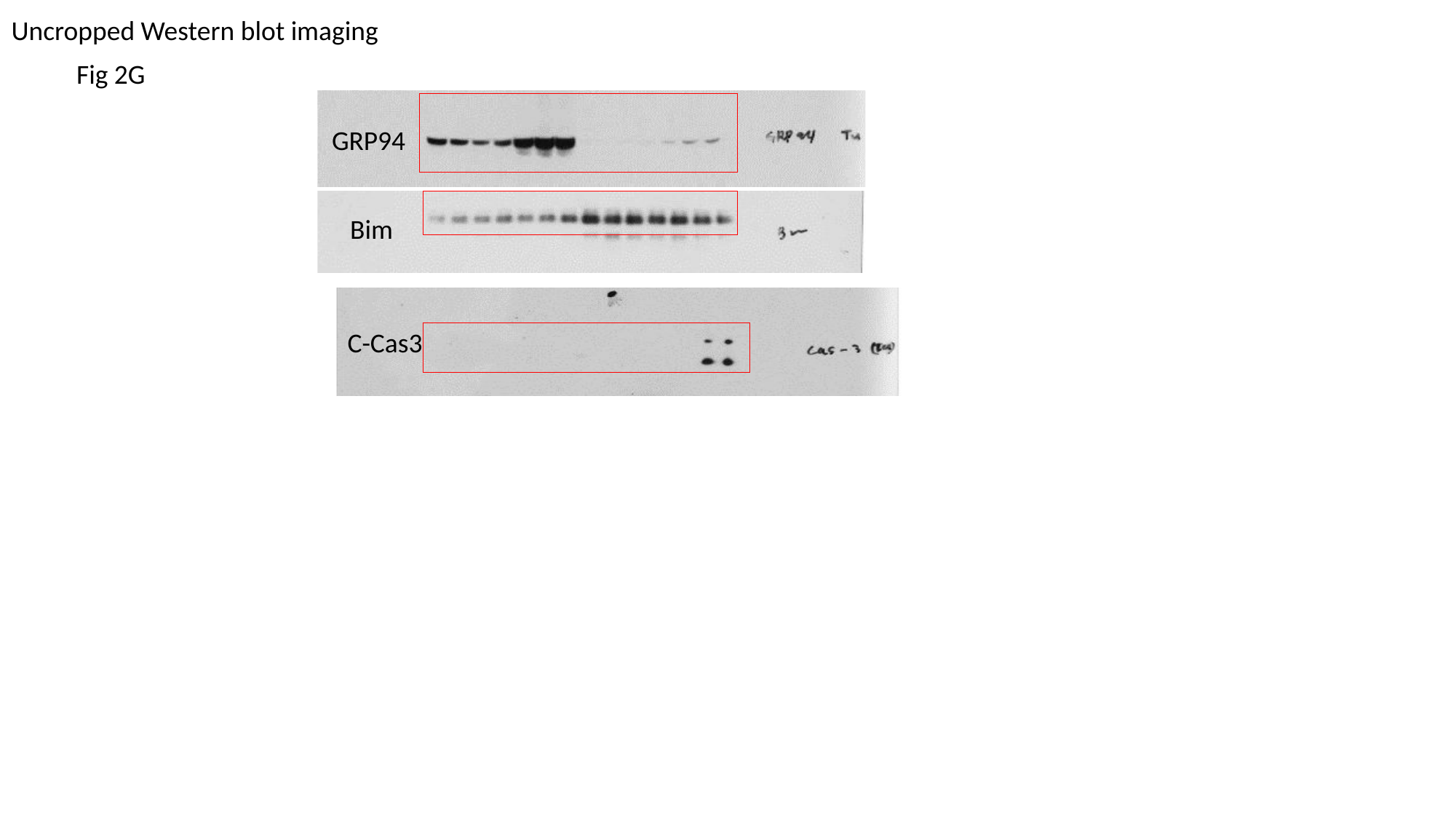

Uncropped Western blot imaging
Fig 2G
GRP94
Bim
C-Cas3

## Slide 3
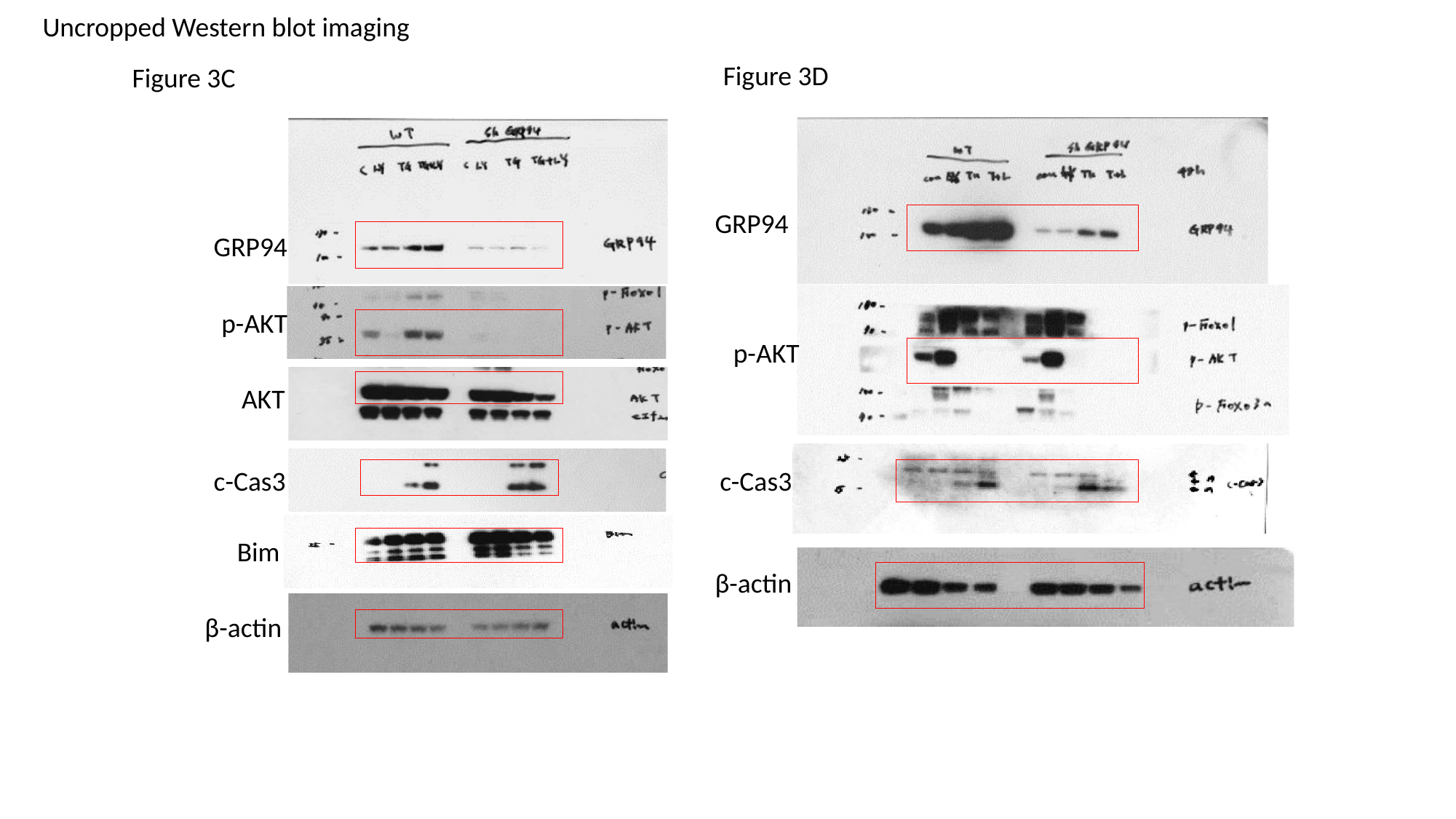

Uncropped Western blot imaging
Figure 3D
Figure 3C
GRP94
GRP94
p-AKT
AKT
c-Cas3
Bim
β-actin
p-AKT
c-Cas3
β-actin

## Slide 4
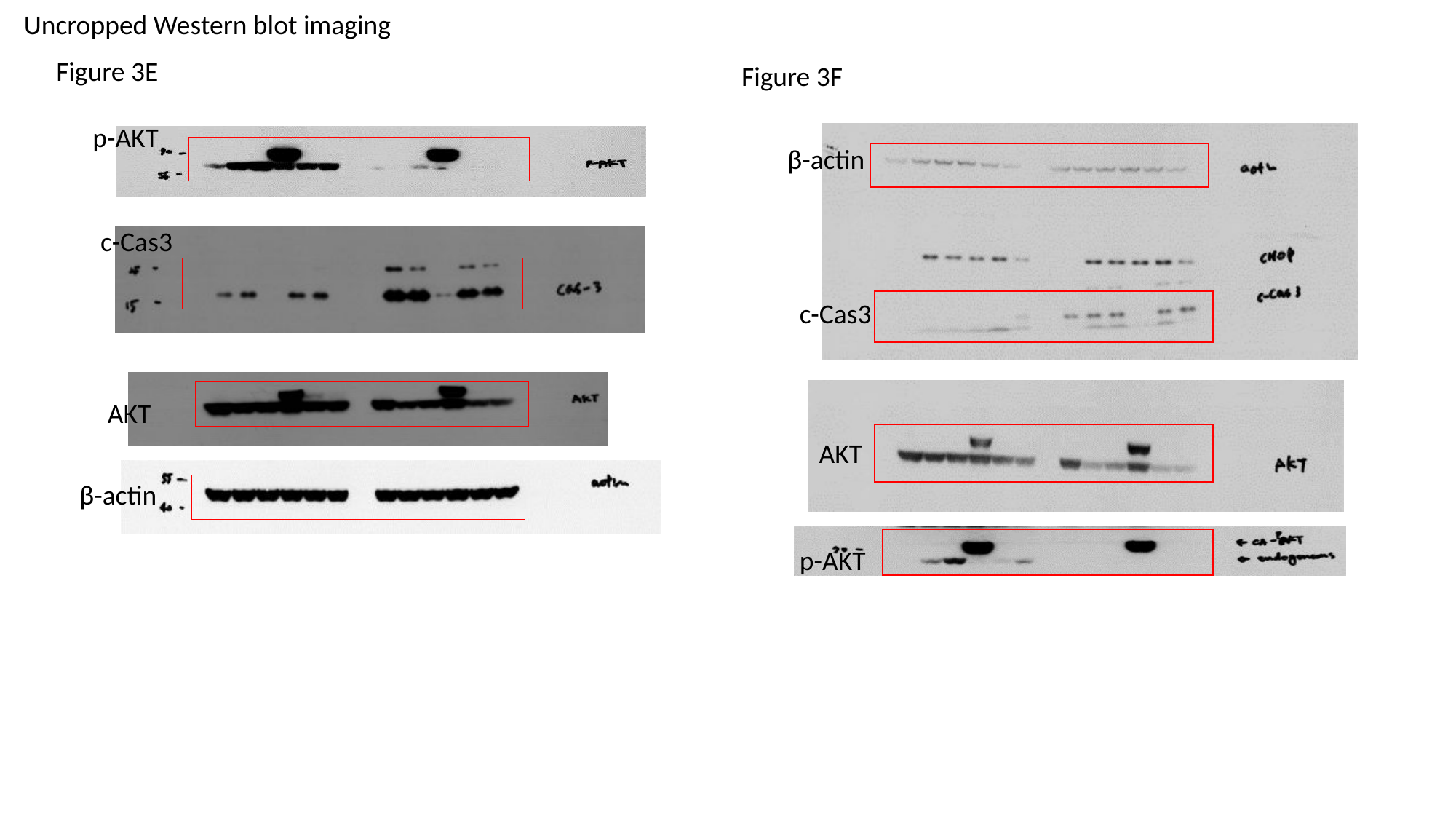

Uncropped Western blot imaging
Figure 3E
Figure 3F
p-AKT
β-actin
c-Cas3
AKT
p-AKT
c-Cas3
AKT
β-actin

## Slide 5
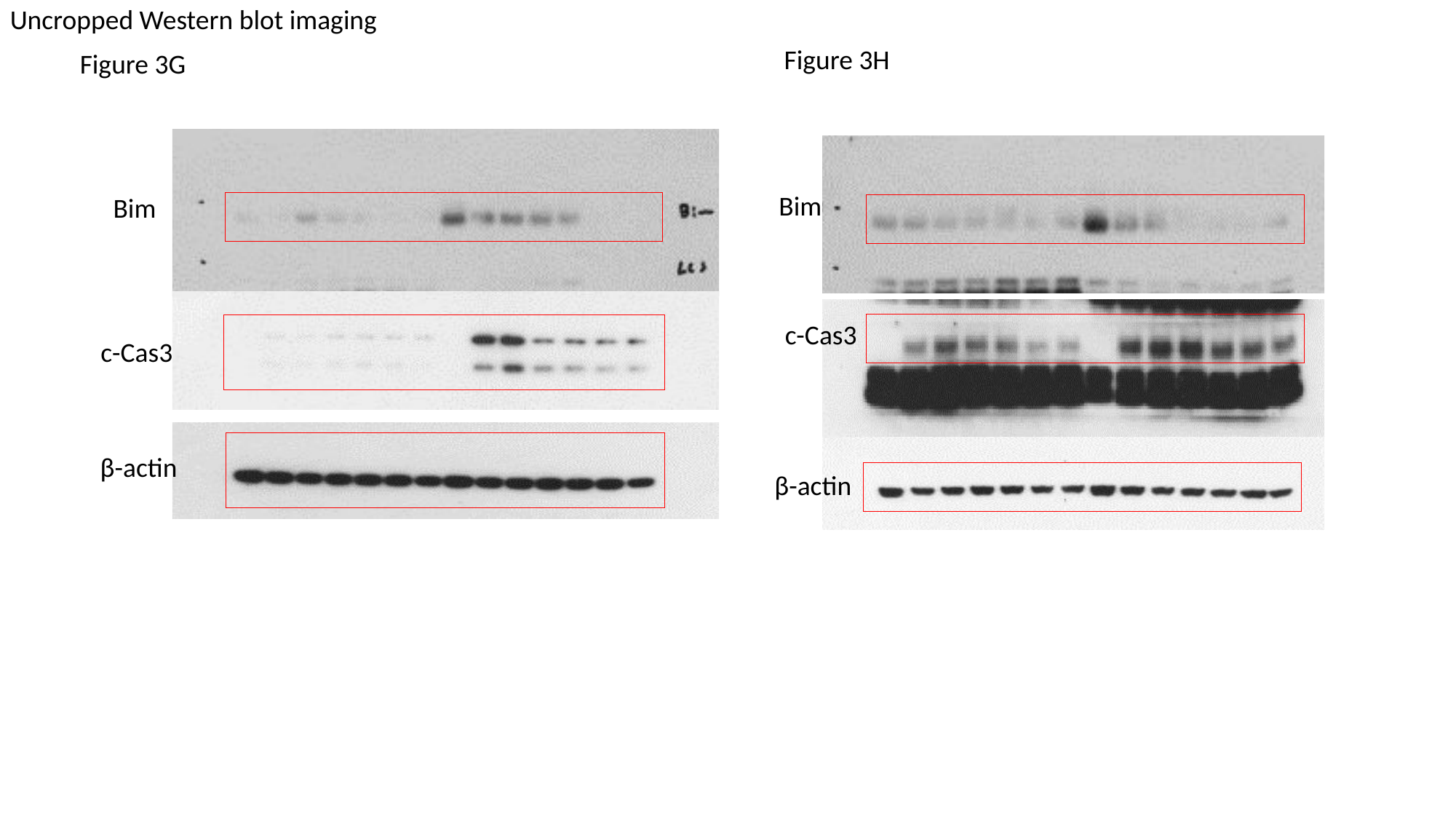

Uncropped Western blot imaging
Figure 3H
Figure 3G
Bim
c-Cas3
β-actin
Bim
c-Cas3
β-actin

## Slide 6
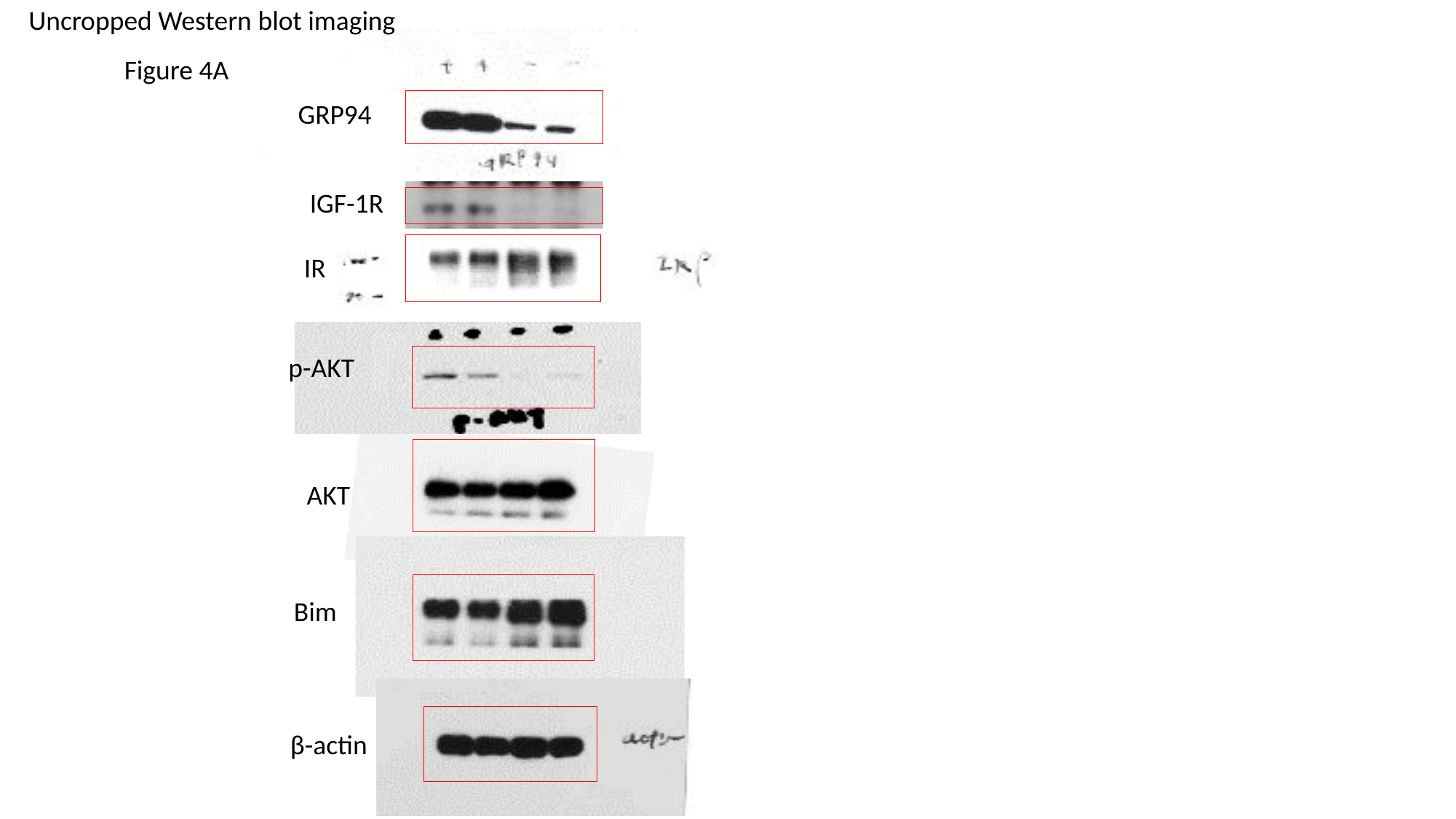

Uncropped Western blot imaging
GRP94
Figure 4A
IGF-1R
IR
p-AKT
AKT
Bim
β-actin

## Slide 7
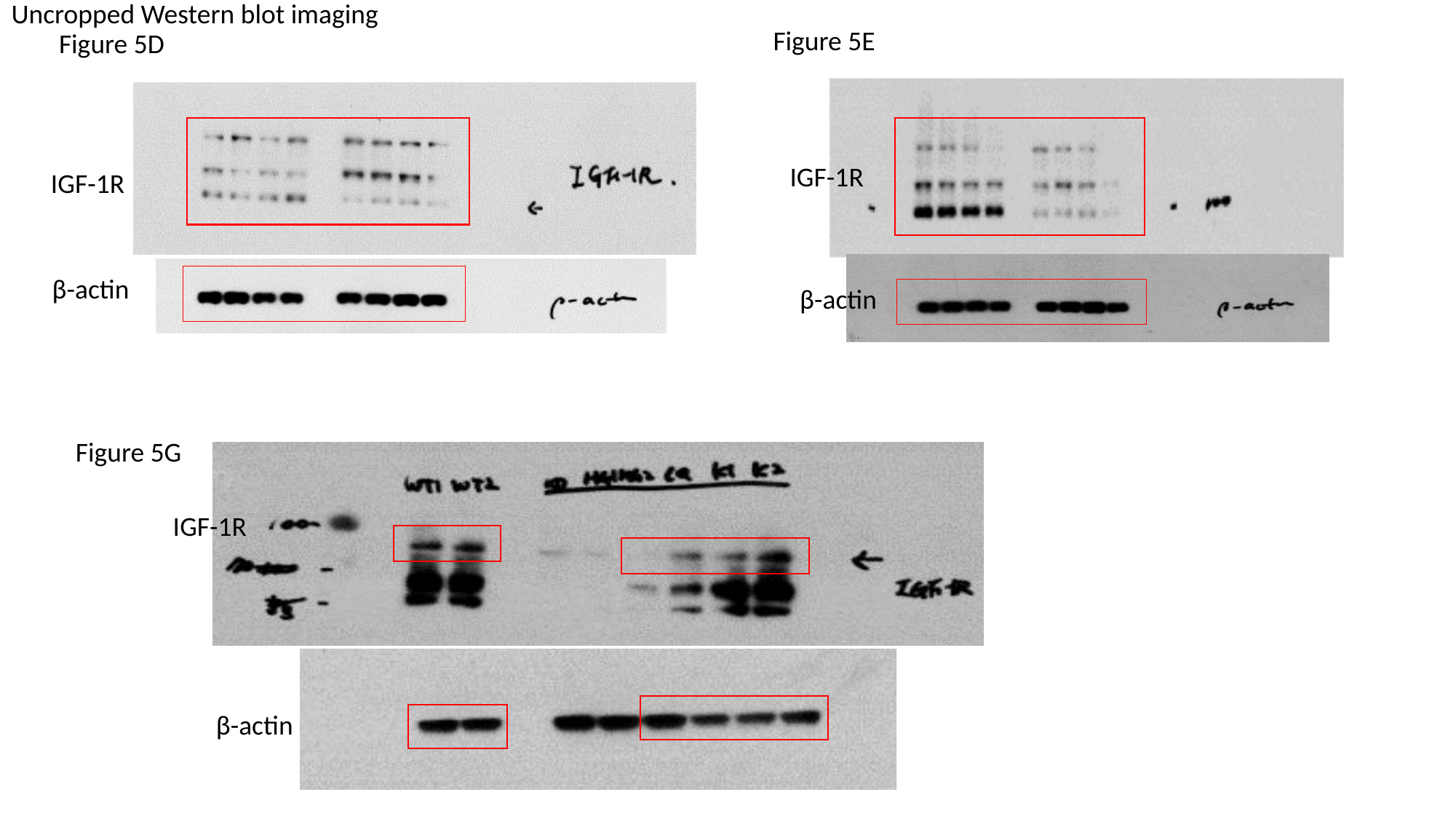

Uncropped Western blot imaging
Figure 5E
Figure 5D
IGF-1R
IGF-1R
β-actin
β-actin
Figure 5G
IGF-1R
β-actin

## Slide 8
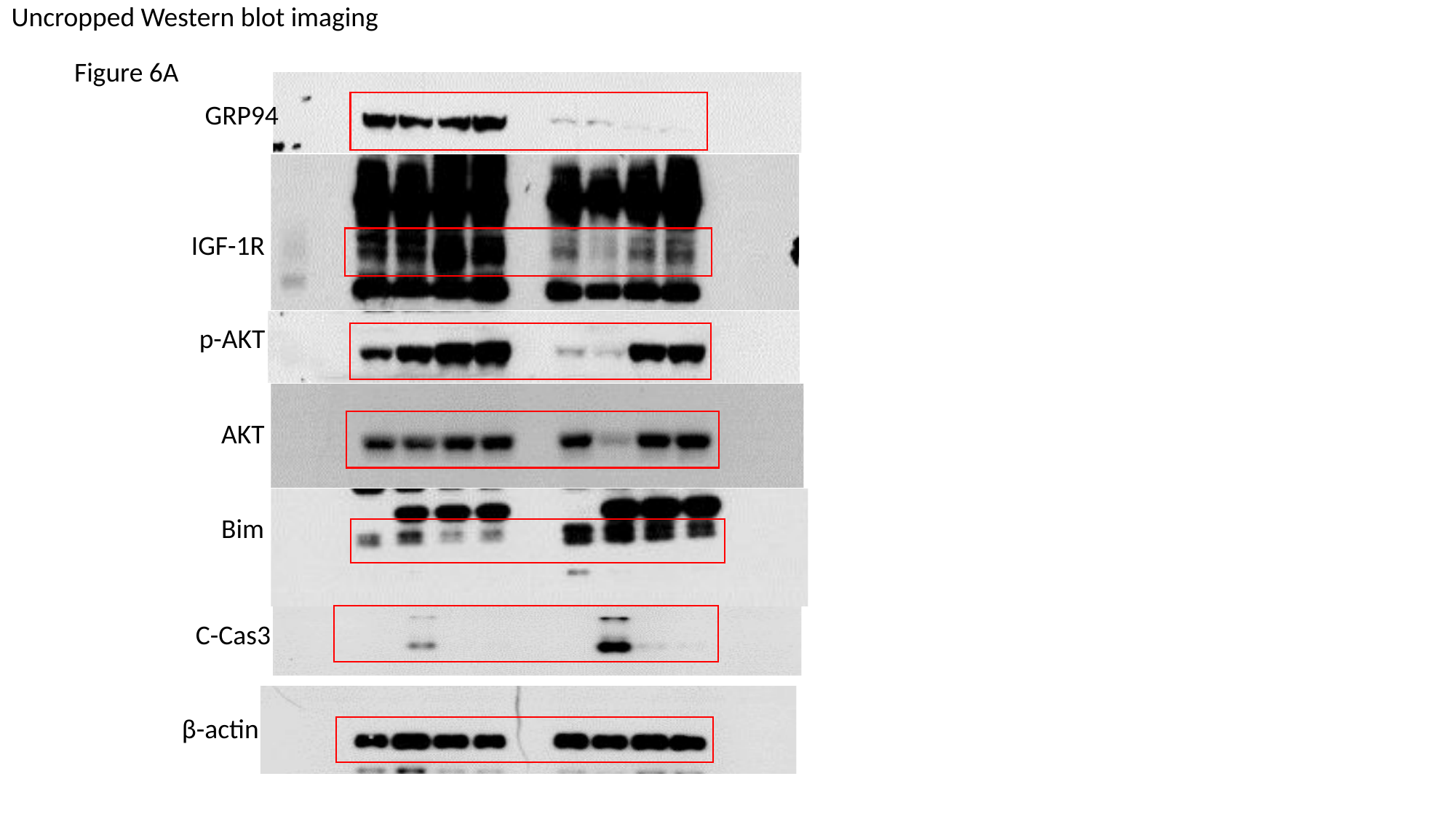

Uncropped Western blot imaging
Figure 6A
GRP94
IGF-1R
p-AKT
AKT
Bim
C-Cas3
β-actin
